# Application of Fibrin Associated with Photobiomodulation as a Promising Strategy to Improve Regeneration in Tissue Engineering: A Systematic Review

**DOI:** 10.3390/polym14153150

**Published:** 2022-08-02

**Authors:** Carlos Henrique Bertoni Reis, Daniela Vieira Buchaim, Adriana de Cássia Ortiz, Simone Ortiz Moura Fideles, Jefferson Aparecido Dias, Maria Angelica Miglino, Daniel de Bortoli Teixeira, Eliana de Souza Bastos Mazuqueli Pereira, Marcelo Rodrigues da Cunha, Rogerio Leone Buchaim

**Affiliations:** 1 UNIMAR Beneficent Hospital (HBU), University of Marilia (UNIMAR), Marilia 17525-160, Brazil; dr.carloshenriquereis@usp.br; 2 Department of Biological Sciences, Bauru School of Dentistry (FOB/USP), University of São Paulo, Bauru 17012-901, Brazil; adrianaortiz@usp.br (A.d.C.O.); simoneortiz@usp.br (S.O.M.F.); 3 Postgraduate Program in Structural and Functional Interactions in Rehabilitation, Postgraduate Department, University of Marilia (UNIMAR), Marilia 17525-902, Brazil; danibuchaim@alumni.usp.br (D.V.B.); jeffersondias@unimar.br (J.A.D.); danielteixeira@unimar.br (D.d.B.T.); elianabastos@unimar.br (E.d.S.B.M.P.); 4 Teaching and Research Coordination of the Medical School, University Center of Adamantina (UniFAI), Adamantina 17800-000, Brazil; 5 Postgraduate Program in Law, University of Marilia (UNIMAR), Marilia 17525-902, Brazil; 6 Graduate Program in Anatomy of Domestic and Wild Animals, Faculty of Veterinary Medicine and Animal Science, University of São Paulo (FMVZ/USP), São Paulo 05508-270, Brazil; miglino@usp.br; 7 Postgraduate Program in Animal Health, Production and Environment, University of Marilia (UNIMAR), Marilia 17525-902, Brazil; 8 Department of Morphology and Pathology, Jundiaí Medical School, Jundiaí 13202-550, Brazil; marcelocunha@g.fmj.br

**Keywords:** tissue regeneration, fibrin, scaffolds, fibrin glue, fibrin sealant, platelet-rich fibrin, photobiomodulation, review, low-level laser therapy

## Abstract

Fibrin, derived from proteins involved in blood clotting (fibrinogen and thrombin), is a biopolymer with different applications in the health area since it has hemostasis, biocompatible and three-dimensional physical structure properties, and can be used as scaffolds in tissue regeneration or drug delivery system for cells and/or growth factors. Fibrin alone or together with other biomaterials, has been indicated for use as a biological support to promote the regeneration of stem cells, bone, peripheral nerves, and other injured tissues. In its diversity of forms of application and constitution, there are platelet-rich fibrin (PRF), Leukocyte- and platelet-rich fibrin (L-PRF), fibrin glue or fibrin sealant, and hydrogels. In order to increase fibrin properties, adjuvant therapies can be combined to favor tissue repair, such as photobiomodulation (PBM), by low-level laser therapy (LLLT) or LEDs (Light Emitting Diode). Therefore, this systematic review aimed to evaluate the relationship between PBM and the use of fibrin compounds, referring to the results of previous studies published in PubMed/MEDLINE, Scopus and Web of Science databases. The descriptors “fibrin AND low-level laser therapy” and “fibrin AND photobiomodulation” were used, without restriction on publication time. The bibliographic search found 44 articles in PubMed/MEDLINE, of which 26 were excluded due to duplicity or being outside the eligibility criteria. We also found 40 articles in Web of Science and selected 1 article, 152 articles in Scopus and no article selected, totaling 19 articles for qualitative analysis. The fibrin type most used in combination with PBM was fibrin sealant, mainly heterologous, followed by PRF or L-PRF. In PBM, the gallium-aluminum-arsenide (GaAlAs) laser prevailed, with a wavelength of 830 nm, followed by 810 nm. Among the preclinical studies, the most researched association of fibrin and PBM was the use of fibrin sealants in bone or nerve injuries; in clinical studies, the association of PBM with medication-related treatments osteonecrosis of the jaw (MRONJ). Therefore, there is scientific evidence of the contribution of PBM on fibrin composites, constituting a supporting therapy that acts by stimulating cell activity, angiogenesis, osteoblast activation, axonal growth, anti-inflammatory and anti-edema action, increased collagen synthesis and its maturation, as well as biomolecules.

## 1. Introduction

The word fibrin, in etymology, derives from the Latin ‘fibre’ (fiber) and –in (chemical substance). It can be defined as a protein formed in blood plasma from the action of thrombin on fibrinogen, being the main component of blood clots (that is, fibrin aggregating produces clots). Wound healing depends entirely on the initial mechanisms of tissue homeostasis. When an injury occurs, the first tissue to respond is blood, as bleeding is a potentially serious risk to the body. There is a cascade of molecular and cellular reactions that lead to the sealing of the vascular lesion with an aggregate of platelets, which stop the hemorrhage by forming a tampon in the injured tissue, triggering the next steps of tissue regeneration. Stable blood clot, containing cross-linked and polymerized fibrin, is essential to prevent bleeding and lead to wound repair after vascular injury [1,2].

Fibrin is a viscoelastic polymer and its mechanical and structural properties as a fibrin scaffold determine its effectiveness in hemostasis and in the development and outcome of thrombotic complications. Fibrin polymerization comprises a series of consecutive reactions, each affecting the final structure of the 3D porous network. Structural features in the fibrin molecule determine the physical properties of clots, and it is important for the blood clot to support arterial flow, clot contraction by platelets, and other dynamic forces [3,4].

The three-dimensional structure of fibrin allows for a series of cellular interactions and provides a temporary matrix in which cells can proliferate, organize, and perform their functions, especially at injured or inflamed sites. Thus, fibrin has been used with the aim of accelerating healing and regeneration in several surgical procedures, especially in medicine in the areas of orthopedics [5,6], neurology [7,8,9], and plastic surgery [10,11], as well as in dentistry in the areas of periodontics [12,13], implantology [14,15], and oral and maxillofacial surgery [16,17].

One of the ways to use fibrin in tissue regeneration is platelet-rich fibrin (PRF) which, unlike platelet-rich plasma (PRP), PRF has a high concentration of fibrin and white blood cells, not platelets. PRP and PRF have the same ability to accelerate the healing of soft and hard tissues by increasing the concentration of growth factors, but PRF acts to release growth factors over a longer period, providing longer lasting benefits, as well as stimulating a faster healing process than PRP [18]. PRF increases the concentration of these factors, among which we can exemplify the platelet-derived growth factor (PDGF), vascular endothelial growth factor (VEGF) and fibroblast growth factor (FGF), factors which help to accelerate neovascularization and cell differentiation [18,19].

Studies also evaluate “fibrin glues” that can be called fibrin adhesive, fibrin sealant or fibrin biopolymer in tissue regeneration [20,21,22,23]. Human fibrin glue is manufactured using two components, one of which is a concentrate of clotting proteins (fibrinogen, fibronectin and Factor XIII) and the other is thrombin, both lyophilized. The first component is reconstituted with an aprotinin solution that inhibits tissue fibrinolysis. Thrombin is mixed with calcium chloride, thus being a grouping of substances participating in hemostasis and wound repair, giving the product properties such as hemostatic action, sealant and biological stimulation, which favor the formation of new tissue matrix [24,25]. In Brazil, a group of researchers from the Center for the Study of Venoms and Venomous Animals (CEVAP/UNESP Botucatu) developed and has been using in several studies, a fibrin sealant without the presence of derivatives from human blood, being totally heterologous, which has components derived from snake venom and fibrinogen from buffalo blood. This sealant, due to its diversity of use, is currently called fibrin biopolymer [8,26].

However, in view of the search for a rapid morphological and functional recovery of the injured tissues, more than one type of therapy can be combined (in this case, a set of therapies complementary to the treatment). One of them is the low-level laser (LLLT), with tissue stimulation properties through red or infrared light with the ability to modulate the repair process, reducing pain, increasing tissue vascularization, promoting an increase in the production of mitochondrial ATP, and a series of biostimulatory effects, which led to the current name of photobiomodulation (PBM) therapy [27,28].

The combined use of fibrin glue with photobiomodulation has shown promising results in the repair of peripheral nerve injuries, being effective in the neurorrhaphy procedure, as well as providing a better quality of axonal regeneration to the interior of the distal stump [29]. In addition, this associated form of therapeutic use has demonstrated the ability to assist in the repair process of bone defects, stimulating angiogenesis and osteoblast proliferation, contributing to the formation of new bone in shorter postoperative periods and in greater volume [30].

However, there are still gaps in explaining the mechanisms of PBM therapy and its effects in combination therapies with fibrin. Therefore, this systematic review was designed from the PICO strategy (P: problem; I: intervention; C: control; O: outcome) [31,32], in order to analyze the relationship between PBM therapy and the use of fibrin compounds, such as PRF and fibrin sealants.

## 2. Materials and Methods

This systematic review was developed in accordance with the Preferred Reporting Items for Systematic Reviews and Meta-Analyses (PRISMA) checklist, as well as other similar research [32,33,34]. For this, PubMed/MEDLINE, Scopus (Elsevier) and Web of Science databases were searched, with a specific search period (1 January 2002–30 April 2022), using the keywords: “fibrin AND low-level laser therapy” and “fibrin AND photobiomodulation”.

With the crossing of keywords, a detailed analysis of the results was carried out, being important in the selection the title and the abstract. From there, the manuscripts were separated into included and excluded according to the eligibility criteria. The authors carried out this process impartially and independently.

- Eligibility Criteria:
  The inclusion criteria were:
  - Therapeutic use of fibrin and PBM therapy as complementary therapy;
  - Studies in humans;
  - Studies in animals;
  - In vivo studies;
  - Case reports;
  - Publications only in English and that allowed full access to the text;
  - Each article included must present data on the PBM protocol.
- The exclusion criteria were:
  - Articles that were duplicated;
  - When the title had no connection to the objective;
  - Did not use fibrin;
  - Did not use photobiomodulation;
  - Used high power laser;
  - Other languages (except English);
  - When access to the full text was not obtained;
  - Incomplete data on the type of fibrin used.
  - Letters to the editor;
  - Review papers;
  - Commentaries;
  - Unpublished abstracts;
  - Dissertations or theses from repositories

Initially, the manuscripts with the title and abstract connected to the topic of the search were verified, with the terms: fibrin and PBM therapy, and then we evaluated and restricted the articles only to the focus of the question in this review. Methodology, the results obtained, and the importance of these results were important to list the selected manuscripts. The selected articles on the topic were carefully read. In addition, two independent reviewers participated in the selection phases, ensuring that the inclusion and exclusion criteria were carefully followed, with the clear objective of minimizing bias.

Data related to the subject of this review were selected and extracted from the manuscripts by independent reviewers, taking into account the characteristics of the individual studies that contributed to their outcome as well as their aggregated results, without the objective of performing a meta-analysis.

**Figure 1:**
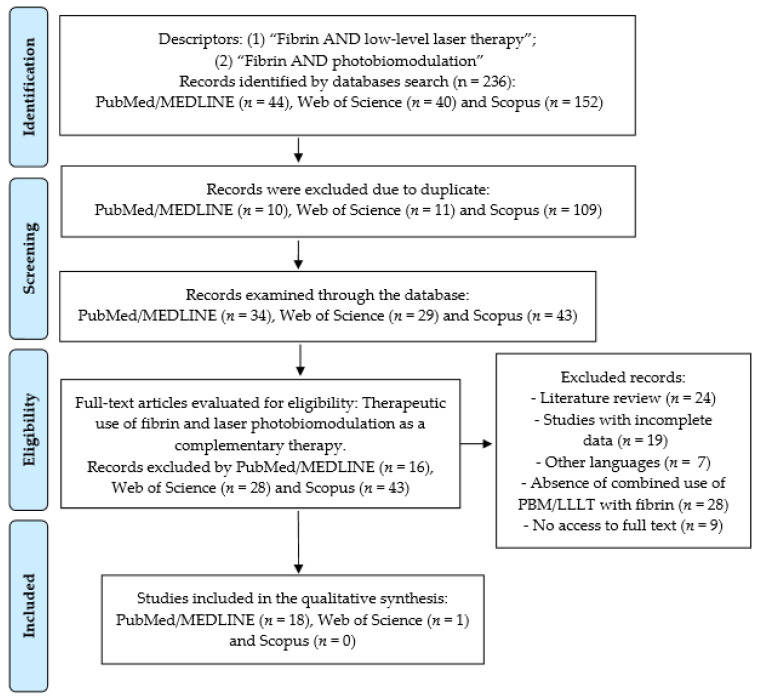
Flow diagram showing study selection.

The selection scheme, according to the PRISMA flow diagram [32,35,36], is shown in Figure 1.

## 3. Results

The bibliography search found 44 articles in the PubMed/MEDLINE database, of which 26 were excluded since they were duplicates or due to inclusion/exclusion criteria. We also found 40 articles in Web of Science and selected 1 article, 152 articles in Scopus and no article selected, totaling 19 articles for qualitative analysis.

**Figure 2:**
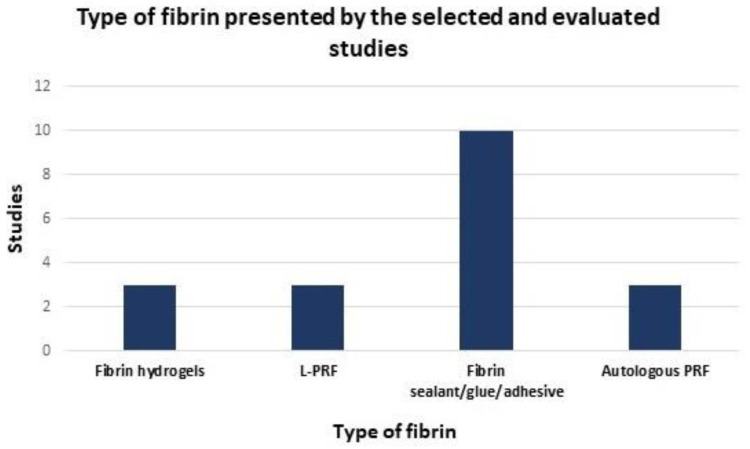
Configurations of fibrin preparations used in tissue regenerative processes. Three studies were used hydrogels or 3D fibrin, 3 with L-PRF, 10 with fibrin sealants (or also called glue, adhesive or biopolymers), and 3 with autologous PRF.

From the studies selected for a detailed description, we can see that, due to their physicochemical characteristics, fibrin compounds are widely used in several areas that mainly involve medicine and regenerative dentistry. In this way, three selected studies were selected in which the researchers used hydrogels or 3D fibrin, 3 with L-PRF, 10 with fibrin sealants (or also called glue, adhesive or biopolymers) and 3 with autologous PRF (Figure 2).

**Figure 3:**
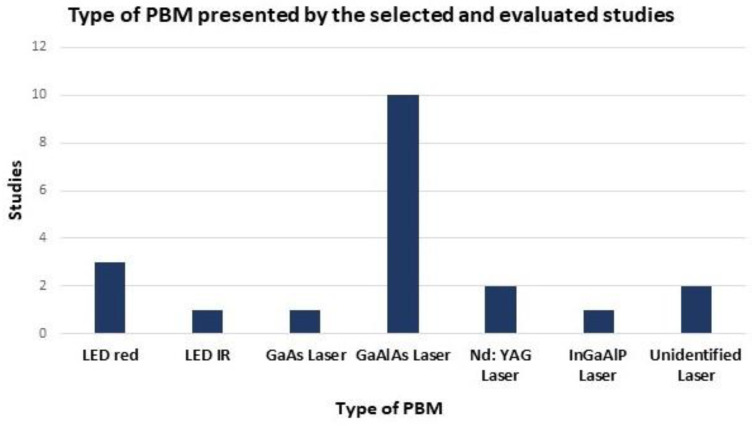
Type of photobiomodulation presented by the selected and evaluated studies. Gallium-Aluminum-Arsenide (GaAlAs) laser that presented greater use in the selected studies in tissue regenerative processes (10 studies). Two studies did not specify the type of PBM used. One study used different types of PBM, therefore considered separately in the data in the figure.

Regarding the results of photobiomodulation, we found (according to the eligibility criteria) three studies that used the red LED (Light Emitting Diode - original apparatus LDM-07 or Repuls Lichtmedizintechnik GmbH, Vienna, Austria), 1 infrared LED (original apparatus LDM-07), 1 GaAs (Gallium-Arsenide) laser (Fisioline; Lumix^®^ C.P.S. Dental Multidiodic laser, Verduno, Cuneo, Italy), 10 GaAlAs (Gallium-Aluminum-Arsenide) laser (Laserpulse IBRAMED^®^, Amparo, Brazil), 2 ND: YAG (neodymium-doped: yttrium aluminium garnet) laser (Fotona, Ljubljana, Slovenia), 1 InGaAlP (Indium-Gallium-Aluminum-Phosphide) laser (MMOptics^®^, São Carlos, Brazil) and two studies did not identify the type of laser used (Figure 3).

**Figure 4:**
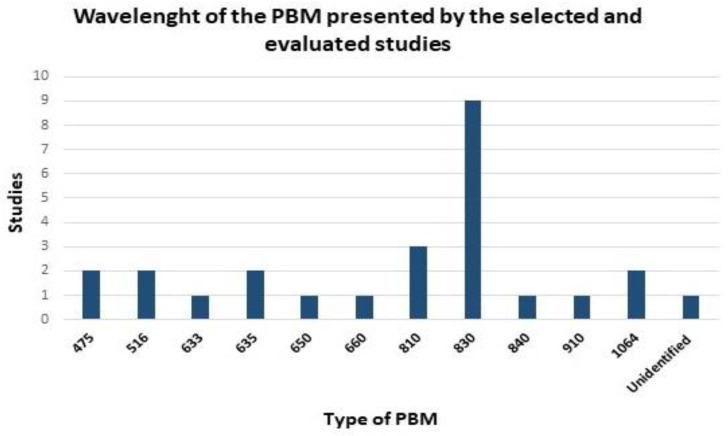
Protocols of PBM. Wavelength (nm) used by the studies included in Table 1. 830 nm that presented greater use in the selected studies in tissue regenerative processes (nine studies). One study did not present the wavelength used. Studies that used different wavelengths were considered separately in the data in the figure.

In the photobiomodulation protocols of the selected studies, when the wavelengths were analyzed, the most used was 830 nm, in nine studies. Then, 810 nm in three studies; 475 nm, 516 nm, 635 nm, and 1064 nm in two studies each; 633 nm, 650 nm, 660 nm, 840 nm, 910 nm with one study each; and one study did not disclose the wavelength used (Figure 4).

The articles selected to compose this review are presented in Table 1.

## 4. Discussion

This systematic review aimed to analyze published research on the association of photobiomodulation therapy, through the use of LLLT or LED, with fibrin scaffolds. The focus was on its use in tissue regeneration, mainly fibrin in the form of PRF and fibrin sealants (glues or adhesives) in order to verify the possible beneficial effects of PBM in three-dimensional fibrin scaffolds.

The initial description of fibrin comes from the classic coagulation cascade, proposed in 1964 by Macfarlane [55] and Davie and Ratnoff [56], documented in several articles. This model referred to as the “cascade” has been proposed to explain the physiology of blood clotting, whereby clotting occurs through sequential proteolytic activation of proenzymes by plasma proteases, resulting in the formation of thrombin, which then breaks down the fibrinogen molecule into fibrin monomers [57]. The fibrin network formed in the clot presents a particularly homogeneous and three-dimensional organization [58]. Furthermore, a progressive polymerization mode means increased incorporation of circulating cytokines in the fibrin meshes (intrinsic cytokines), providing an increase in the lifespan of these cytokines. Thus, cytokines are kept in situ for a convenient period when the scar cells begin to remodel the matrix, at which time they need to be stimulated to participate in the reconstruction of the injured site [59,60].

Due to its characteristics and properties, fibrin has been used in several areas, one of which is tissue regeneration in medical and dental procedures. Among the forms presented, in this review, three studies were selected for qualitative analysis that used hydrogels or 3D fibrin, 3 with L-PRF, 10 with fibrin sealants and 3 with autologous PRF (Figure 2). The production of autologous platelet concentrates (APCs) occurs by centrifuging the patient’s own blood, injecting isolated plasma, which is rich in growth factors. In tissue regeneration, two generations of APCs have been used: PRP, which are first generation, produced by double-spin centrifugation of blood; and PRF, the second generation, produced by single-spin centrifugation and has the fibrin matrix network intact. The effectiveness of platelet concentrates in promoting wound healing and tissue regeneration is at the center of recent academic discussion [61].

In a preclinical study, using LLT, PRF and Nano-HA nanohydroxyapatite graft (Fisiograft^®^, Ghimas, Italy) as variables, Hemaid et al., (2019) observed that the use of PRF + NanoHA mix results in an increase in bone fill and density regarding the radiographic outcomes in induced periodontal intrabony defects in rabbits, and LLLT may improve the results [44]. To prepare the PRF, five-milliliter blood samples were collected from each rabbit and then centrifuged at 30,000× *g* RPM for 15 min. The PRF was separated into two pieces; one was used as a membrane and the other was cut into pieces to be added with Fisiograft^®^ plus Nano-HA.

**Figure 5:**
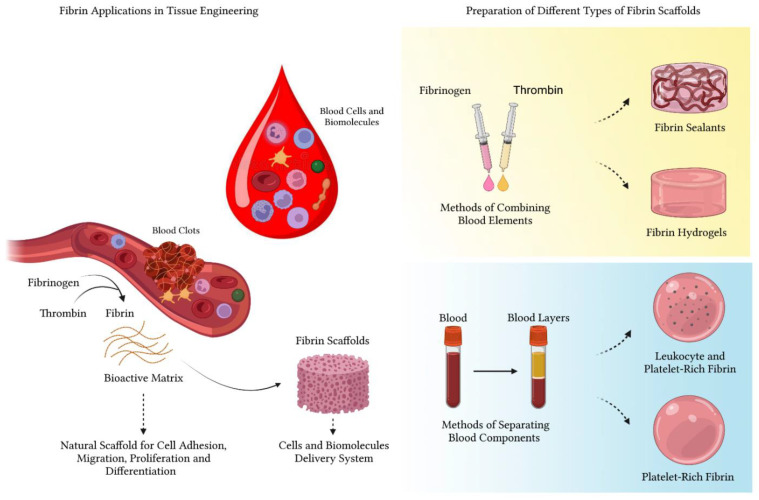
Schematic overview of fibrin applications in tissue regeneration. Fibrin is a plasma protein formed by the action of thrombin on fibrinogen, and constitutes a natural component of the blood coagulation cascade. The three-dimensional structure of the fibrin matrix serves as a natural scaffold that favors cell adhesion, migration, proliferation and differentiation, in addition to favoring the interaction with biomolecules and growth factors. Thus, fibrin has been used to promote tissue regeneration in various segments of medicine, in the form of sealants, hydrogels, PRF or L-PRF.

However, the study by Doan et al., (2020) the clinical applicability of the combination of autologous concentrated growth factors (CGF) and photobiomodulation (PBM) was made. Lateral sinus windows were created using piezoelectric surgery (PES) and the dental implants were concurrently fixated and wrapped with autologous fibrin (AF) rich CGF. Wound sites PBM treatment using a multiwave locked system laser. Bovine demineralized freeze-dried bone (Bio-Oss^®^, Chatswood, Australia) and hydroxyapatite and calcium triphosphate (Genoss^®^, Seoul, Korea) were incorporated into CGF for grafting. The application of AF offers benefits such as being a safe procedure, easy to perform and low cost [54] (Figure 5).

Three studies used L-PRF, developed in 2001 in France by Dr. Joseph Choukroun [62], during the production technique an attempt was made to accumulate platelets and release cytokines in a fibrin clot. This technique does not require anticoagulants, bovine thrombin or any other gelling agent, unlike other platelet concentrates; it is simply centrifuged natural blood without additives [58,63]. When using L-PRF, there are different methods and protocols in its production. In a pre-clinical study, in critical defects in the calvaria of rats, two methods of obtaining the concentrate were analyzed, by means of high (L-PRF) or low speed (A-PRF) centrifugation. The L-PRF and A-PRF groups had significantly higher bone volume and newly formed bone area than the control group (clot only) and reduced bone porosity values, but with no significant difference between them in the histomorphometric and microtomographic analysis. Therefore, L-PRF and A-PRF potentiated the healing of critical defects, and high and low-speed centrifugation protocols did not produce PRF matrices with different biological impacts on the amount of new bone formation [64].

Leukocyte and platelet-rich fibrin (L-PRF) also have been used widely for bone tissue engineering. L-PRF has the potential to, in cases of bone loss, collaborate in osteogenic differentiation, increase osteoblast proliferation, tissue neovascularization and lower risk of local contamination [65,66]. The three studies in Table 1 that used L-PRF were combinations with PBM for the treatment of jaw osteonecrosis, all with good and promising results for use in the treatment of this type of bone disease [38,45,48]. Among the growth factors stored in platelets, which are essential for the tissue repair, are PDGF. Also present are VEGF-A, transforming growth factor-beta (TGF-β1), FGF–2, epidermal growth factor (EGF), hepatocyte growth factor (HGF), and insulin-like growth factor–1 (IGF–1) [67]. It should be taken into account the fact that L-PRF does not use the inclusion of anticoagulant and activating agents (CaCl_2_) to obtain the platelet concentrate. The inclusion of these agents and activators, in addition to hard-centrifugation (≥210 g), can affect the amount and quality of platelet recovery and growth factor release, which can significantly influence healing behavior compared to natural fibrin clotting [68].

Three studies were used hydrogels or 3D fibrin [37,41,42], associated with PBM, being incorporated into the fibrin matrix endothelial cells [41], stromal vascular fraction (SVF) and mesenchymal stromal cells (MSCs) isolated from human gingival mucosa [37]. These studies agree that photobiomodulation combined with fibrin enhances the improvement of results, collaborating in cell and vascular proliferation.

Fibrin sealants were most commonly used in combination with PBM, in ten studies [29,39,40,43,47,49,50,51,52,53]. One of the studies used the fibrin sealant derived from human plasma (Tisseel Lyo^®^ (Baxter Healthcare Ltd., Norfolk, UK) [43] and the others a heterologous fibrin sealant (HFS). This bioproduct (HFS) is composed of a thrombin-like enzyme purified from the venom of *Crotalus durissus terrificus* snake and a cryoprecipitate rich in fibrinogen extracted from *Bubalus bubalis* buffaloes (produced by CEVAP/UNESP—Center for the Study of Venoms and Venomous Animals, Botucatu, Brazil). HFS has several advantages in its use, such as a fast production process, low cost, potential to act as a scaffold for stem cells [69,70,71] and biomaterials [50,72], and as a new drug delivery system [73]. Its indications are in medical, veterinary and dental practice, due to the possibility of personalized formulation and replacement of conventional sutures. Considering all of the properties described for this bioproduct, which go beyond the adhesive capacity, the name “sealant” was reconsidered, and it has recently been called “fibrin biopolymer” [74,75].

**Figure 6:**
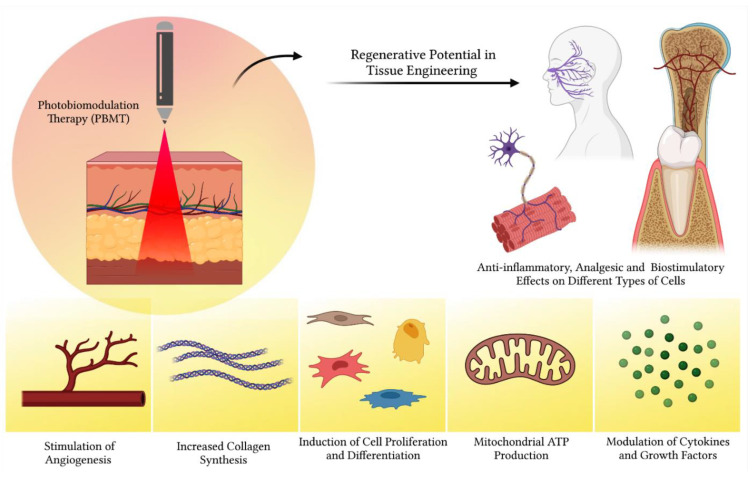
Schematic overview of beneficial properties of photobiomodulation therapy in regenerative medicine. The application of laser therapy favors angiogenesis, collagen synthesis, mitochondrial ATP production, cytokines and growth factors synthesis, in addition to inducing cell proliferation and differentiation. Additionally, photobiomodulation therapy has anti-inflammatory, analgesic and biostimulating effects, acting mainly in the initial stages of tissue healing.

In order to improve the tissue repair process, studies in the area of regenerative science seek the association of different therapies to accelerate and improve morphological recomposition and faster functional recovery. Among these conjunctions, light-based therapies, such as the use of low-power lasers and LEDs, have expanded their use in clinical and pre-clinical practices. The laser consists of a pure and well-defined color, while the LED can display different shades of colors at once. Therefore, the laser is a monochromatic light (only a well-determined color) and the LED is a polychromatic light, being able to present all of the shades of a specific color. Currently called photobiomodulation (PBM), consists of the application of light (Laser or LED) with therapeutic effect for tissue modulation (activation or inhibition). It has important potentialities such as angiogenesis and neovascularization [76], increase in collagen production [77], increase in muscle regeneration and decrease in its atrophy [78], favors nerve regeneration [9,79], increases cartilage production [80], and decreases inflammation, edema and pain [81] (Figure 6).

In the studies selected for Table 1, according to the eligibility criteria, three used the red LED, 1 infrared LED, 1 GaAs laser, 10 GaAlAs laser, 2 ND: YAG laser, 1 InGaAlP laser and two studies did not identify the type of laser used (Figure 3). Handler et al. (2021) carried out a study to investigate the effects of photobiomodulation at wavelengths of 660 nm (Aluminium-gallium-indium-phosphide laser, AlGaInP) and 830 nm (Arsenide-Gallium-Aluminum laser, AsGaAl) at different numbers of application points on the healing of open wounds in mice. Photobiomodulation with total energy of 3.6 J was applied at 1, 4, 5 and 9 points for 14 days. When comparing the photobiomodulation wavelength, the 830 nm (AsGaAl) groups were more effective, and the groups irradiated at 5 points stand out, which showed improvement in macroscopic analysis and epidermis thickness, increased number of vessels and lower number of fibroblasts on the 14th day after the skin lesion [82].

Regarding the wavelength, the most used was 830 nm, in nine studies. Then 810 nm in three studies; 475 nm, 516 nm, 635 nm, and 1064 nm in two studies each; 633 nm, 650 nm, 660 nm, 840 nm, and 910 nm with one study each; and one study did not disclose the wavelength used (Figure 4). A study conducted by Ma et al. (2018), to determine the effect of low-level laser therapy (LLLT) on diabetic wound healing and confirm its effect on the activity of healthy human fibroblasts, used PBM with an 830 nm (IR) wavelength, 635 nm (Red) and 635 nm + 830 nm (FX) with the same fluency of 60 J/cm^2^. Irradiation in the FX and IR groups showed a significant increase in fibroblast proliferation and collagen synthesis compared to the control and RED groups. However, there was no significant difference in collagen synthesis and fibroblast proliferation between the FX group and the IR group. These data allowed the authors to conclude that healthy human fibroblasts showed better cell proliferation and collagen synthesis when irradiated at the wavelength of 635 nm + 830 nm or 830 nm [83].

The use of LED photobiomodulation is more recent than laser therapy. Current research advances in the evaluation of the separate or combined use of the two therapies in tissue repair. Doses ranging from 0.1 to 10 J/cm^2^ and wavelengths from 405 to 1000 nm promote therapeutic benefits in tissue regeneration. Ranges of light energy sources, from lasers to LEDs, have been used and have specific advantages and limitations. There is no consensus on standardized treatment parameters such as wavelengths, therapeutic outcomes and doses, which limits direct comparison and clinical protocol recommendation [84,85,86,87,88,89,90].

**Figure 7:**
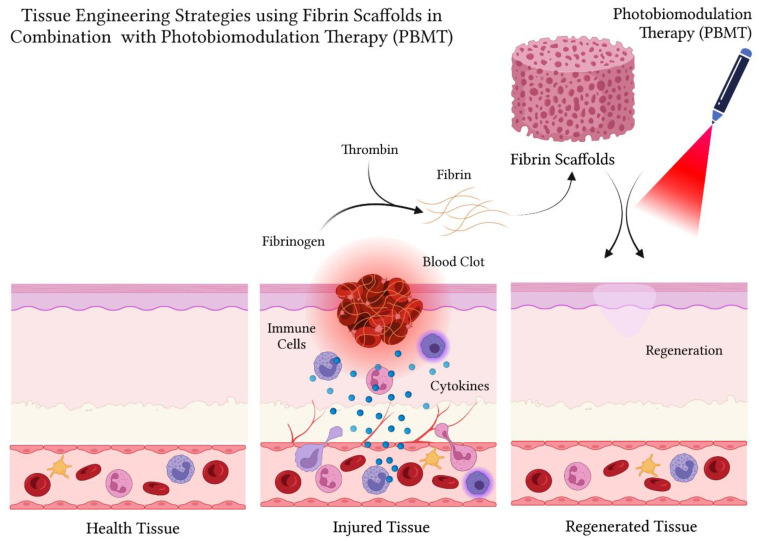
The application of fibrin combined with photobiomodulation therapy constitutes a promising strategy to favor regeneration in tissue engineering. Fibrin applied to the injury site forms a bioactive matrix that exerts a hemostatic effect, in addition to favoring interactions between cells and biomolecules. Photobiomodulation constitutes a coadjuvant therapy that acts by stimulating cell activity, angiogenesis and the synthesis of collagen and biomolecules. Thus, the application of fibrin associated with photobiomodulation therapy may have a beneficial effect, accelerating tissue healing.

The use of combined therapies that involve the use of fibrin associated with photobiomodulation therapy has shown to be a promising strategy to favor the regeneration of injured tissues with better quality and less time. When fibrin is applied to the lesion site, it forms a bioactive matrix in the microenvironment that exerts a hemostatic effect, in addition to favoring interactions between cells and biomolecules (Figure 7). These effects, added to those of PBM, constitute a supporting therapy that acts by stimulating cell activity, angiogenesis, and the synthesis of collagen and biomolecules [49,91,92,93,94,95,96,97,98,99,100].

In this review, among preclinical studies, the most researched association of fibrin and photobiomodulation was the use of fibrin sealants in bone or nerve injuries. In clinical studies, the association of PBM with medication-related treatments osteonecrosis of the jaw (MRONJ). All experimental protocols concluded that the association is effective; promoting a more effective repair of lesions, in a shorter period of time and with effectiveness that can reinforce the indication of its use. In peripheral nerves, PBM therapy accelerated morphological and functional nerve repair. In bone tissue [51], PBM allowed for an improvement in the formation of new bone, with a more organized deposition of collagen fibers in the defect area [50]; and in osteonecrosis of the jaw, PBM may effectively contribute to MRONJ management [38,45,101].

In this way, we can see that few studies used the association fibrin + PBM, but given the good results, the technique is promising, with the potential to collaborate in tissue repair. The difficulty in comparing the different types of PBM can be considered as a limitation, due to the different protocols reported in the experiments. Therefore, protocols with favorable results are generally standardized and reused by the same researchers in an attempt to reduce this limitation. In addition, the scarcity of randomized clinical trials in the scope of this review can also be considered a limitation.

## 5. Conclusions

This review was designed and carried out with the objective of analyzing studies, both clinical and pre-clinical, that used the association of photobiomodulation and fibrin. This association occurs with the purpose of tissue regeneration, in the search for its possible beneficial effects on morphophysiological and functional rehabilitation. The fibrin matrix, with its three-dimensionality, is a natural scaffold, which enables events that favor the repair of injured tissues, which is desired in tissue engineering procedures, through adhesion, migration, proliferation, and cell differentiation, in addition to contributing to the interaction with biomolecules and local tissue growth factors.

In the findings of this study, it can be shown that PBM contributed to improve tissue regeneration that used fibrin composites as scaffolds, constituting an important adjuvant therapy that acts by stimulating cell activity, angiogenesis, osteoblastic activation, axonal growth, anti-inflammatory and anti-edema action, increased collagen synthesis and its maturation, as well as biomolecules. More studies should be carried out in order to seek standardization in PBM protocols, in the same way that new fibrin concentrates will be developed with the same objective of recovering injured organs and tissues.

## Figures and Tables

**Table 1:** Articles that were selected for detailed analysis, following the eligibility criteria.

Reference (Database)	Type of Laser/LED (Manufacturer)	Wavelength (nm) and Output Power (mW)	Power Density (mW/cm^2^)	Energy Density (J/cm^2^)	Objective	Fibrin	Intervention	Outcome/Results	Conclusions
Bikmulina et al., 2020 [37] (PubMed)	LED light red and infrared (IR) (Original apparatus LDM-07)	Red: 633 IR: 840 and Red: 160 ± 20 IR: 320 ± 40	Red: 1.8 ± 0.2 IR: 3.6 ± 0.4	Red and IR: 2.2 ± 0.2	Evaluation of PBM therapy for cell stimulation in hydrogels	Mesenchymal stromal cells (MSCs) obtained from human gingiva mucosa were encapsulated in fibrin (hydrogels)	A single exposure was made to low-intensity light, both red and infrared. After three days of culture, the physiological activity and viability of the cells were verified	The authors observed a dependence on cell viability in relation to the concentration of gel-forming proteins and the thickness of the hydrogels	Infrared light can be indicated for stimulation of MSCs proliferation and metabolism, in hydrogels with thicknesses of up to 3 mm
Tenore et al., 2020 [38] (PubMed)	Red and Infrared Gallium-Arsenide laser (GaAs) (Fisioline; Lumix^®^ C.P.S. Dental Multidiodic laser)	Three wavelengths: 650, 810, 910 and G1: total power of 600 mW; G3 total power of 1100 mW	-/-	-/-	To evaluate the effect of three different protocols on the healing outcome in patients with established medication-related osteonecrosis of the jaw (MRONJ)	Leukocyte- and platelet-rich fibrin (L-PRF)	G1 was treated with antibiotic therapy, surgery, L-PRF and PBM; G2 with antibiotic therapy and surgery; G3 with antibiotic and PBM	There was no significant association between MRONJ results and location, stage, duration of drug treatment, diabetes, smoking, corticosteroid therapy, underlying disease, sex, and chemotherapy history at three and six months	The combination of antibiotic therapy, L-PRF, surgery and PBM can effectively contribute to the treatment of MRONJ
Buchaim et al., 2015 [29] (PubMed)	Gallium-Aluminum-Arsenide (GaAlAs) (Laserpulse IBRAMED^®^, Amparo, Brazil)	830 and 30	-/-	4	To analyze whether the fibrin adhesive allows, through end-to-side neurorrhaphy, the collateral growth of axons without an epineural window of the vagus nerve into a sural nerve graft and whether laser therapy contributes to the regeneration process	Fibrin glue derived from snake venom	Experimental Group (EG; *n* = 12 rats), sural nerve graft was coapted to the vagus nerve with fibrin glue; and experimental group laser (EGL; *n* = 12 rats), EG + LLLT and control group (CG; *n* = 8 rats), the intact sural nerve was collected	There was sprouting of axons from the vagus nerve into the autologous graft in the EG and EGL, and in the CG all of the dimensions measured were better, with a significant difference in relation to the EG and EGL, except for the area and thickness of the myelin sheath, which showed a significant difference only in relation to the EG	LLLT potentiates nerve regeneration and fibrin glue provided conditions for axonal regeneration in peripheral nerve injuries
de Oliveira Gonçalves et al., 2016 [39] (PubMed)	GaAlAs (Laserpulse IBRAMED^®^, Amparo, Brazil)	830 and 30	258.6	6	To evaluate the effects of LLLT on an autogenous bone graft integration process stabilized with a new heterologous fibrin sealant (NHFS)	Heterologous fibrin sealant	Autogenous bone graft from rat calvaria, removed from the right parietal bone, with a 5 mm osteotomy, was adhered on the left side with fibrin sealant; groups: autogenous Fibrin graft (AFG) and autogenous fibrin graft laser (AFGL), with the same procedures as the AFG, plus LLLT	The bone regeneration process was not complete, with new bone tissue partially integrating the graft into the recipient bed, with some areas of connective tissue. Morphometrically, minor interfaces occurred in the AFGL group, with significant differences in all analyzed periods	LLLT stimulated bone neoformation and improved the process of integration of autogenous bone graft
Buchaim et al., 2017 [40] (PubMed)	GaAlAs (Laserpulse IBRAMED^®^, Amparo, Brazil)	830 and 30	258.6	6.2	To analyze the efficacy of LLLT on quantitative, qualitative and functional aspects in the facial nerve regeneration	NHFS derived from snake venom	Suture experimental (SEG) and fibrin experimental (FEG) groups, the buccal branch of the facial nerve was sectioned, end-to-end epineural suture on the right side, and a NHFS on the left side; laser suture experimental (LSEG) and laser fibrin experimental (LFEG) groups, the same procedures as SEG and FEG with the addition of LLLT; control group (CG), facial nerve intact	LLLT resulted in a significant increase in the density and number of new axons. The LSEG and LFEG presented better scores in functional analysis in comparison with the SEG and FEG	Both repair techniques were effective in promoting axonal growth and LLLT improved these results, in addition to accelerating the functional recovery of whiskers
Rohringer et al., 2017 [41] (PubMed)	LED lamps were provided by Repuls Lichtmedizintechnik GmbH, Vienna, Austria	Pulsed LED light of either 475 nm (blue), 516 nm (green), 635 nm (red) or remained unstimulated (control)	Peak irradiance intensity of 80 mW/cm^2^ on all LED devices; average irradiance intensity of 40 mW/cm^2^	Dose 24 J/cm^2^ (daily)	To compare the effects of PBM using light-emitting diodes (LED) with different wavelengths on endothelial cells in vitro	3D fibrin matrices and fibrin gels	Migration and proliferation tests were performed in 2D and 3D. 3D fibrin gel co-culture model with human umbilical vein endothelial cells (HUVEC) and adipose-derived stem cells (ASC) was used to analyze early vasculogenic effects, continuous stimulation of LLLT, after one week of culture	Stimulation with green and red LED light increased 3D migration and proliferation of HUVEC. HUVEC also had greater potential for 2D migration with green light stimulation. Blue light was ineffective	Green light, in several parameters, has been shown to be more potent in stimulating endothelial cell migration and proliferation than red light
Priglinger et al., 2018 [42] (PubMed)	LED lamps were provided by Repuls Lichtmedizintechnik GmbH, Vienna, Austria	Pulsed LED light 475 nm (blue), 516 nm (green), 635 nm (red)	All LED devices had a peak irradiance intensity of 80 mW/cm^2^	Fluence of 24 J/cm^2^	To analyze the effects of green, blue and red light (RL) emitted by LEDs directly on freshly isolated SVF and analyzed cell phenotype, cell number, viability, ATP content, LDH cytotoxicity and proliferation, but also osteogenic, adipogenic and pro-angiogenic differentiation in vitro	3D fibrin matrices	Pulsed blue (475 nm), green (516 nm) and RL (635 nm) from LEDs applied on freshly isolated Stromal Vascular Fraction (SVF)	LLLT increased, compared to untreated cells, the colony-forming unit fibroblast assay with RL. The frequency of colony forming cells was not affected. LLLT with green light and RL resulted in a better potential to form vascular tubes by SVF compared to untreated cells when grown in 3D fibrin matrices	LLLT has beneficial effects in relation to SVF cell proliferation and vascularization potential. LLLT may represent a good method for clinical practice in activating SVF cells
Pomini et al., 2019 [43] (PubMed)	GaAlAs (Laserpulse IBRAMED^®^, Amparo, Brazil)	830 and 30	258.6	6	In rat calvaria (critical size defect—CSD), to evaluate the scaffold formed by a fibrin sealant (FS) plus xenograft associated with PBM therapy	Tisseel Lyo^®^ (Baxter Healthcare Ltd., Norfolk, UK)	CSD in calvaria, 36 rats: 4 groups: BC (*n* = 8), defect with blood clot; FSB (*n* = 10), FS and xenograft; BC^PBMT^ (*n* = 8), blood clot and PBM; FSB^PBMT^ (*n* = 10), FS, xenograft, and PBM	Bone neoformation was observed in all groups, limited to the defect margins. In the FSB group, new bone increased between periods (4.3 ± 0.46 to 6.01 ± 0.32), but with lower volume when compared to the FSB^PBMT^ (5.6 ± 0.45 to 10.64 ± 0.97)	The biocomplex formed by the xenograft plus FS associated with the PBM therapy had a positive effect on the new bone formation
Hemaid et al., 2019 [44] (PubMed)	Diode Laser Gallium-Aluminum-Arsenide (GaAlAs)	810 and 100	-/-	46.8	To observe and compare the combined use of LLLT (810 nm), PRF and NanoHA in the healing of induced intraosseous periodontal defects	Autologous platelet-rich fibrin (PRF)	Sixteen defects in rabbits divided in four groups: laser irradiated control (CL); Control non-treated (C); PRF + NanoHA graft treated group and laser irradiated (NanoHA-Graft + PRF + L)	NanoHA-Graft + PRF + L showed significantly higher bone density in relation to the other groups	The best form of treatment was the combined use of LLLT + PRF + NanoHA as it presented the best results in the formation of new bone
Sahin et al., 2020 [45] (PubMed)	Nd: YAG laser (Fotona, Ljubljana Slovenia)	1064 and 1250	-/-	-/-	To analyze the surgical procedures used to prevent the development of MRONJ after dentoalveolar surgery in patients who received bisphosphonates	Leukocyte and platelet-rich fibrin (L-PRF)	Sixty-three surgeries were performed on forty-four patients taking bisphosphonate. Procedures: performed dentoalveolar surgical; antibiotics; fill the socket with L-PRF; LLLT (Nd: YAG laser)	There were no intercurrences until cure. Complete mucosal healing occurred in all patients within one month with no long-term failures	The surgical protocol demonstrates promising results for the protection of MRONJ after performing dentoalveolar surgeries
Thalaimalai et al., 2020 [46] (PubMed)	Diode laser	810 and 500	-/-	-/-	To evaluate the combined effect of LLLT and PRF, in site modulated intra-bony defects, which were accessed using a simplified papilla preservation flap (SPPF), on the periodontal disease	Autologous platelet-rich fibrin	Thirty patients with intra-bony defects (2 groups, *n* = 15 each). There was SPPF access at test group (TG) sites and defects received intramedullary penetration (IMP) after debridement, followed by LLLT and PRF grafting. In the control group (CG), the defects were accessed with SPPF and grafted only with PRF	TG showed a clinically relevant increase in mean probing pocket depth reduction, clinical attachment level gain, and radiographic bone fill compared to the CG, six months post-intervention	Together, LLLT with PRF caused an improvement in clinical and radiographic results within modulated intraosseous defects
Della Coletta et al., 2021 [47] (PubMed)	GaAlAs (Laserpulse IBRAMED^®^, Amparo, Brazil)	830 and 30	258.6	6.2	To evaluate the effects of PBM therapy on the guided bone regeneration process (GBR) in defects in the calvaria of rats filled with biphasic calcium phosphate (BCP) associated with fibrin	Fibrin biopolymer (FB)	Thirty Wistar rats: BMG, defects filled with biomaterial and covered by membrane; BFMG, biomaterial and fibrin biopolymer (FB) covered by membrane; and BFMLG, biomaterial and FB covered by membrane and biostimulated with PBM	There was more evident bone growth in the BFMLG, in addition to a progressive increase in new bone tissue in all groups, with a significant difference in the BFMLG, whose group presented greater bone neoformation in the periods of 14 and 42 days, followed by BFMG and BMG	PBM has been shown to be effective in improving and accelerating the GBR process when associated with BCP and FB
Sahin et al., 2021 [48] (PubMed)	Nd: YAG laser (Fotona, Ljubljana Slovenia)	1064 and 1250	-/-	-/-	To analyze the surgical technique described in the treatment of advanced stages of MRONJ patients	Autologous L-PRF concentrate	Twnty-one patients affected by Stage 2-3 MRONJ were treated with ultrasonic piezoelectric for bone surgery, with necrotic bone removing, L-PRF and LLLT	Two patients, who were Stage 3, had delayed healing at 1 month after surgery. Complete mucosal healing occurred in all patients in the third month	The surgical protocol shows promising results for surgical management of advanced stages of MRONJ patients
de Freitas Dutra Júnior et al., 2021 [49] PubMed	Indium-Gallium-Aluminum-Phosphide laser (InGaAlP) (MMOptics^®^, São Carlos, Brazil)	660 and 40	1000	6	To verify, in tendon injuries, the action of the new heterologous fibrin biopolymer (HFB) associated or not with PBM	Heterologous fibrin biopolymer	Partial transection calcaneus tendon (PTCT) was performed in 84 rats divided into 4 groups: control (CG); HFB; PBM; HFB + PBM. HFB was applied immediately after PTCT, while PBM started 24 h after injury and continued every 24 h for 7, 14 and 21 days.	It can be noted that the reduction of edema was effective in the treatment groups when compared to the CG. In the periods of 14 and 21 days, PBM had a better repair process compared to GC	The HFB and PBM treatments, associated or isolated, promoted a reduction in the edema volume, favoring the repair process. HFB alone contributed more in promoting the tendon repair process
Buchaim et al., 2022 [50] (PubMed)	GaAlAs (Laserpulse IBRAMED^®^, Amparo, Brazil)	830 and 30	258.6	6.2	To analyze the effects of PBM on CSD filled with xenogenic bone substitute associated with HFB	Heterologous fibrin biopolymer (HFB)	CSD in 36 Wistar rats, four groups: BC and BC-PBM (controls) with defects filled by a clot (without or with PBM); XS and XS-PBM, filled with biocomplex Bio-Oss^®^ + HFB. PBM was applied transoperatively and continued three times a week	BC-PBM and XS-PBM had a higher density of the bone neoformation in relation to the groups without PBM. Significant vascular proliferation and new bone deposition around the XS particles were observed in the animals which biocomplex (XS and XS-PBM)	PBM allowed an improvement in none neoformation, with a more organized deposition of collagen fibers. Biocomplex favored the permanence and insertion of the particulate biomaterial in bone defect
Rosso et al., 2017 [51] (PubMed)	GaAlAs (Laserpulse IBRAMED^®^, Amparo, Brazil)	830 and 30	260	6.2	To evaluate the action of PBM on lesions of the facial nerve repaired with the end-to-side technique or coaptation with a NHFS	New Heterologous Fibrin Sealant	Thirty-two rats, five groups: control (CG); experimental suture (ESG) and experimental fibrin (EFG) groups, end-to-side sutured to the zygomatic branch on the right side of the face or NHFS on the left side; experimental suture laser (ESLG) and experimental fibrin laser (EFLG) groups, with PBM	There was a significant difference in the fiber nerve area between the EFG and the EFLG. There was also faster functional recovery of the whisker movement in the ESLG and EFLG, where PBM was used, with results closer to the CG	Photobiomodulation with LLLT accelerated functional and morphological nerve repair, in both techniques
Rosso et al., 2020 [52] (PubMed)	GaAlAs (Laserpulse IBRAMED^®^, Amparo, Brazil)	830 and 30	258.6	6	To evaluate the action of PBM on rat tibial defect filled with biomaterial of the lyophilized bovine bone matrix (BM) associated or not with HFB	Heterologous fibrin biopolymer (HFB)	Thirty rats, three groups. A noncritical bone defect of 2 mm was produced. Four Groups: (1) BM + PBMT; (2) BM + HFB; (3): BM + HFB + PBM. In Groups 1 and 3 the animals were submitted to intraoperative PBM and every 48 h until the period of euthanasia	Statistical difference in bone neoformation between Groups 3 and 2 (26.4% ± 1.03% and 20.0% ± 1.87%, respectively) at 14 days and 42 days (38.2% ± 1.59% and 31.6% ± 1.33%, respectively). In 42 days there was presence of new bone with mature characteristics	The combined use of PBM with HFB and BM contributed to the process of reconstruction of non-critical bone defects
Buchaim et al., 2016 [53] (PubMed)	GaAlAs (Laserpulse IBRAMED^®^, Amparo, Brazil)	830 and 30	258.6	6	To evaluate the effects of LLLT in the repair of the buccal branch of the facial nerve with two techniques: coaptation with HFS and end-to-end epineural suture	Heterologous fibrin sealant (HFS)	Forty-two rats, five groups: (1) control (CG), facial nerve (buccal branch) was collected without lesion; (2) experimental suture (EGS) and experimental fibrin (EGF) groups: end-to-end suture on the right side and HFS on the left side; (3) experimental suture laser (EGSL) and experimental fibrin laser (EGFL): plus LLLT	Axonal growth occurred in the distal stump of the facial nerve in all groups. The morphological aspect was similar to the GC fibers, with the majority of myelinated fibers. In the last period of the experiment, the EGSL presented the best results, being closer to the CG, in all measurements performed, except in the axon area	Laser therapy showed better results in facial nerve regeneration, being an effective technique to stimulate the repair process of peripheral nerve injuries
Doan et al., 2020 [54] (Scopus)	MLS laser (ASA laser, Vicenza, Italy)	-/-	-/-	1.27	Two clinical cases with piezoelectric surgery (PES), concentrated growth factors (CGF) and PBM, used in the search to increase the formation of new blood vessels and tissue repair after maxillary sinus lift surgeries with dental implants	Autologous concentrated growth factors (CGF)	The lateral sinus windows were created using PES. The implants were inserted in the same surgery and wrapped with CGF. A laser treatment of PBM was performed at the site, applied in the apical, buccal, lingual, coronal, mesial and distal regions of the surgical wound	Vascular budding and wound closure was observed after the first day. New bone formation was detected in the enlarged maxillary sinuses next to the implants, through radiographs and cone-beam computed tomography	PBM, PES, and CGF promoted the formation of new vessels, favored the approximation of the edges, closing the wound and reducing edema and bleeding. In addition, there was less postoperative pain, less use of analgesics and speech impairment, without trismus

## Data Availability

Not applicable.
